# Psychometric Analysis and Cross‐Cultural Comparisons of the Italian and English Sense of Humor Scale Parallel Version Short Form

**DOI:** 10.1111/sjop.70049

**Published:** 2025-12-15

**Authors:** Chloe Lau, Willibald Ruch, Sonja Heintz, Lena C. Quilty, Francesco Bruno, Donald Saklofske, Francesca Chiesi

**Affiliations:** ^1^ Centre for Addiction and Mental Health Toronto Ontario Canada; ^2^ Department of Psychiatry, Schulich Medicine and Dentistry Western University London Ontario Canada; ^3^ Department of Psychology University of Zurich Zurich Switzerland; ^4^ School of Psychology University of Plymouth Plymouth UK; ^5^ Department of Psychiatry University of Toronto Toronto Ontario Canada; ^6^ Department of Human and Social Sciences, Faculty of Social and Communication Sciences Universitas Mercatorum Rome Italy; ^7^ Department of Psychology University of Western Ontario London Ontario Canada; ^8^ Department of Neuroscience, Psychology, Drug, and Child's Health (NEUROFARBA), Section of Psychology University of Florence Florence Italy

**Keywords:** humor, Italian, laughter, personality, psychometric

## Abstract

The Sense of Humor Scale parallel version short form (SHS‐PSF) is a novel self‐report measure aimed at describing personality traits related to enjoyment of humor, laughter, verbal humor, humor under stress, humor in everyday life, and laughing at oneself. The present study recruited Italian (*N* = 298) and Canadian (*N* = 910) participants to complete the Italian and English versions, respectively, to assess the measurement properties of the newly translated Italian SHS‐PSF together with Canadian results. The bifactor and six‐factor models show more optimal fit indices than the one‐factor model, albeit insufficient incremental validity indices. Based on Samejima's graded response model, item discrimination parameters ranged from 0.32 to 2.58 (median = 1.24), with 27 of 29 items showing moderate to very high discrimination parameters. Conditional reliability estimates reveal accurate measurements across the latent continuum. Four items had uniform differential item functioning (DIF) when comparing the Italian and English SHS‐PSF (McFadden's pseudo *R* ^2^ > 0.035 or *β* > 0.10). The Italian SHS‐PSF has insufficient‐to‐acceptable psychometric properties. Cross‐language measurement evaluation comparisons suggest significant biases in 4 of 29 items using conservative DIF approaches.

**Summary**

- The study translated and evaluated the Italian Sense of Humor Scale—Parallel Version Short Form (SHS‐PSF) using Italian adults (*N* = 298) and compared measurement properties with Canadian undergraduates (*N* = 910).
- Bifactor and six‐factor models fit better than a one‐factor model, though incremental fit indices (CFI/TLI) were insufficient, indicating only partial support for the theorized structure; item loadings were acceptable across all six humor skills.
- Most items showed moderate to very high discrimination (27/29 items), with strong conditional reliability across the humor continuum. Two items showed low discrimination and may contain measurement noise.
- Italian subscales correlated as expected with cheerfulness, extraversion, optimism, lower bad mood, and life satisfaction, supporting convergent/discriminant validity; enjoyment of humor was the weakest subscale.
- Metric invariance but not scalar invariance was achieved, and four items showed uniform DIF between Italian and English versions, indicating cultural or linguistic response biases that limit direct score comparisons across languages.

## 1 Introduction

A sense of humor is a valued social trait linked to both physical and psychological well‐being (Ruch 2008; Ruch and Hofmann 2017). Humor can be understood as social warmth and skillful humorous behavior (Craik et al. 1996), with cognitive and emotional aspects serving as coping mechanisms in challenging situations (Martin and Lefcourt 1983). In personality assessment, humor‐related traits capture amusement, mirth, and exhilaration (Ruch et al. 1996; Ruch 1997; Ruch and Hofmann 2012), expressed as everyday behaviors (e.g., sarcasm, cheerfulness, humor under stress) or peak performance (e.g., humor creativity and production). These traits shape how people appreciate, understand, and express humor across contexts, with emotional and cognitive aspects likely universal (Ruch and Hofmann 2012). Individual differences influence how easily people laugh and create humor (Ruch et al. 1996, 1997). Humor‐related traits are associated with enhanced creativity, reduced negative emotions, and greater life satisfaction (Lau et al. 2019; Lau et al. 2024; Ruch and Hofmann 2017), and humor training has been shown to foster these qualities in clinical and workplace settings (Falkenberg et al. 2011; León‐Pérez et al. 2021; Tagalidou et al. 2018; Wellenzohn et al. 2016).

McGhee (1996) proposed a multifaceted model of humor, emphasizing high playfulness and low seriousness as key for recognizing and responding to life's incongruities. His training program targeted six humor habits, enjoying humor, laughing, using verbal humor, finding humor in everyday situations, laughing at oneself, and using humor under stress, through progressively challenging exercises. This structured approach increased humor and life satisfaction among participants, with close acquaintances also perceiving them as more humorous (Ruch et al. 2018).

The sense of humor scale (SHS) was developed to assess the six humor habits in research, assessment, and intervention (McGhee 1996; Ruch and Carrell 1998). It measures humor comprehension, appreciation, and production, though some subscales (laughter, enjoyment of humor) showed lower reliability (Ruch and Carrell 1998). The SHS factors correlated strongly with playfulness and seriousness in American and German samples, and while Wrench and McCroskey (2001) suggested a general factor, Lydon and McDermut (2021) found that a bifactor model provided the best fit. The scale has demonstrated good internal consistency and test–retest reliability across diverse samples, though some items appear outdated, underscoring the need for revision (Lydon and McDermut 2021).

To improve the SHS, Ruch and Heintz (2018) developed the SHS parallel version, combining new and original items. It revealed six distinct factors with unique variance beyond a global humor factor. Whereas the original SHS showed lower reliability for enjoyment of humor (*α* = 0.56) and laughter (*α* = 0.65), the parallel version improved reliability (*α* = 0.71–0.88). Most fit indices were acceptable, though the CFI remained modest (0.84–0.88) across six‐factor, hierarchical, and bifactor models.

Heintz et al. (2021) developed a 29‐item short form of the SHS‐P (SHS‐PSF), which showed strong psychometric properties, including high internal consistency and factorial, criterion, and construct validity, with expected links to life satisfaction. Items were selected based on content coverage, distinctiveness, and exploratory structural equation modeling. The SHS‐PSF demonstrated metric invariance across gender and culture (English‐ and German‐speakers), and the six humor skills explained more variance in humor outcomes and life satisfaction than the total score, supporting their incremental validity (Heintz et al. 2021; Ruch and Heintz 2018). No studies have yet evaluated humor training in Italian contexts; given prior evidence of its benefits, translating the SHS could support intervention research in Italy (Ruch et al. 2018). The SHS‐P framework captures cognitive, emotional, and behavioral aspects of humor, offering a comprehensive tool for research.

In terms of construct validity, SHS scores correlate positively with extraversion and openness but not with other Big Five traits (Lydon and McDermut 2021). Cheerfulness, a facet of extraversion, shows stronger predictive validity for humor‐related emotions, thoughts, and behaviors (Ruch and Hofmann 2012), overlapping by 74% with the six humor skills and showing strong network associations (Lau et al. 2022a; Ruch and Carrell 1998; Ruch and Heintz 2018). SHS scores also converge with humor as a character strength, self‐ and peer‐rated socially warm humorous behavior, and more frequent and prolonged laughter (Müller and Ruch 2011; Ruch and Carrell 1998; Soury and Devillers 2014; Wrench and McCroskey 2001). The six humor habits correlate positively with life satisfaction (Ruch and Heintz 2018), and comedians score higher than other groups on the SHS total and five of six subscales, except enjoyment of humor (Lydon and McDermut 2021). These findings support the SHS's convergent and predictive validity, though limited evidence exists for the short form, underscoring the need for further research to confirm its reliability and validity. A shorter measure would reduce fatigue while enabling assessment of related constructs during humor training (Ruch and Heintz 2018).

Although the SHS has shown strong psychometric properties in German and English, it has not yet been translated into Italian, limiting its use in humor research there (e.g., Bischetti et al. 2021; Lau et al. 2020; Lau et al. 2023; Ruch and Forabosco 1996). Ruch and Forabosco (1996) found that Italians reported greater appreciation of sexual humor but lower funniness ratings compared to Germans, though both groups could differentiate resolvable from non‐resolvable incongruities. Recent studies have examined humor in Italy during COVID‐19 (Bischetti et al. 2021; Canestrari et al. 2021) and evaluated humor self‐reports without focusing on cross‐language measurement evaluations (Burro et al. 2022; Dionigi et al. 2022; Sirigatti et al. 2014). Few studies have tested invariance: Lau et al. (2020) replicated the three‐factor structure of humor temperament (cheerfulness, seriousness, bad mood) but found metric, not scalar, invariance between Italian and English respondents. Similarly, Lau et al. (2025) showed that while gelotophobia, gelotophilia, and katagelasticism measures performed well in Chinese and English, the Italian katagelasticism scale was reliable only about one standard deviation above the mean. Italians were also significantly less likely than Canadians to endorse items such as “I often tell jokes,” with large effect sizes. These findings underscore the need to validate humor self‐reports in Italian, as cultural differences may shape responses.

To increase access to humor assessment and training in Italy, it is essential to adapt the SHS‐PSF into Italian for use alongside other translated humor measures (e.g., Lau et al. 2020). A validated Italian version would expand opportunities for humor assessment and intervention research within a cross‐language measurement evaluation framework. This study examines differential responding in the SHS factor structure between Italian and Canadian participants. With growing interest in cross‐language measurement evaluation (e.g., Heintz et al. 2020), translating and validating such measures is crucial for advancing global humor research and practice.

### 1.1 The Present Study

This study had three aims: (1) to assess the reliability and validity of the newly translated Italian SHS‐PSF, (2) to evaluate its concurrent and discriminant validity with related constructs, and (3) to investigate cross‐language measurement equivalence. First, internal consistency and validity of the Italian SHS‐PSF were examined through scale scores, distributional characteristics (mean, SD, skewness, kurtosis), confirmatory factor analyses, and item response theory (IRT) parameters. No prior work has evaluated SHS IRT parameters, which clarify item discrimination (i.e., identifying measurement noise) and category thresholds along the latent continuum. Establishing where the SHS provides precise measurement, and identifying items with noise, may guide future revisions.

Second, criterion validity was tested by correlating SHS‐PSF factors with personality traits, humor temperament, optimism, stress, and life satisfaction. Prior studies have linked humor to life satisfaction (Ruch and Heintz 2018). We expected positive associations with cheerfulness, optimism, and life satisfaction, and negative associations with stress, bad mood, and seriousness, consistent with McGhee's (1996) theory that humor skills enhance positive emotions and reduce seriousness.

Finally, multigroup CFA and differential item functioning analyses assessed cultural invariance between the Italian and English SHS‐PSF. These analyses determine whether findings from both versions can be meaningfully compared across cultures.

## 2 Methods

### 2.1 Participants and Procedure

#### 2.1.1 Sample 1

Sample 1 consists of 298 Italian speakers (68.8% female, 29.5% male, and 0.6% nonbinary or other), aged between 19 and 72 years (*M* = 33.67; SD = 15.63), who were recruited for an online study. The sample was recruited using snowball sampling, beginning with undergraduate participants who subsequently invited family members and friends to take part. The vast majority of participants (98.7%) reported Italian as their first language, with 63.8% residing in central Italy. Participants' relationship statuses were as follows: single (30.9%), in a relationship (31.5%), cohabiting or married (33.9%), divorced (3.4%), and widowed (0.3%). Regarding educational background, participants reported the following: primary school certificate or not reported (0.5%), lower secondary school (2.3%), upper secondary school (66.4%), university degree/diploma (12.8%), and post‐graduate degree (17.4%). The study received approval from the Non‐Medical Research Ethics Board from the University of Florence before data collection began.

#### 2.1.2 Sample 2

Sample 2 consisted of 910 English‐speaking undergraduate students (71.1% female, 28.6% male, and 0.3% identifying as another gender) from a Canadian university who completed the SHS‐P online via Qualtrics, a web‐based survey platform. Participants' ages ranged from 16 to 40 years (*M* = 18.30, SD = 1.25). Initially, there were 951 responses, but 41 were excluded due to failing an attention check item (i.e., “please select neutral”), resulting in 910 valid responses. The majority of participants identified as Asian/Pacific Islander (42.1%) or European White (39.7%). Participants were informed that the study aimed to explore the relationships between personality traits and well‐being. Undergraduate students received course credit for completing the survey. Participation in both studies was voluntary, and participants were debriefed afterward. The study received approval from the Non‐Medical Research Ethics Board from the University of Western Ontario before data collection began.

### 2.2 Measures

#### 2.2.1 Sense of Humor

The SHS‐PSF includes 29 items designed to assess six humor skills: enjoyment of humor, laughter, verbal humor, finding humor in everyday life, laughing at yourself, and humor under stress (Heintz et al. 2021; Ruch and Heintz 2018). Enjoyment of humor (4 items; e.g., “on the internet, I am looking for content that amuses me.”) measures the appreciation of humorous content that individuals may actively seek out. Laughter (3 items; e.g., “I have a good belly laugh many times each day”) captures the enjoyment and lightheartedness experienced in the moment. Verbal humor (6 items; e.g., “I often tell jokes.”) pertains to the creation and sharing of humorous content, including learning new jokes and expressing them spontaneously. Finding humor in everyday life (6 items; e.g., “I often find humor in things that happen at home”) involves recognizing fun or lighthearted aspects in various situations and shifting from a serious to a playful perspective. Laughing at yourself (5 items; “If someone makes a joke about me, I can laugh about it.”) reflects the ability to appreciate and make lighthearted fun of oneself. Humor under stress (5 items; “Even in difficult situations I don't lose my sense of humor”) denotes the ability to find and use humor in challenging situations, including reframing them in a humorous way.

Participants rate their responses on a seven‐point Likert scale from 1 (*strongly disagree*) to 7 (*strongly agree*). Following standard test translation procedures, two bilingual psychologists translated each item from English to Italian, ensuring lexical difficulty and content equivalence were preserved (Beaton et al. 2000; Hambleton and Lee 2013). A third bilingual psychologist then back‐translated the items into English, and all items were reviewed to confirm that the content remained consistent. No significant issues were found with the Italian translations. Items are found in Supporting Information S1.

#### 2.2.2 Humor Temperament

The Italian version of the State Trait Cheerfulness Inventory—Trait Version short form consists of 30 items that assess humor temperament traits including cheerfulness, seriousness, and bad mood (Ruch et al. 1996; Italian version; Lau et al. 2020). These traits are measured using a four‐point Likert scale (1 = *strongly disagree*; 4 = *strongly agree*). Previous research has shown that this version has structural validity, strong item discrimination parameters, and both convergent and discriminant validity (Lau et al. 2020).

#### 2.2.3 Personality

The Ten Item Personality Measure (TIPI; Gosling et al. 2003; Italian version; Chiorri et al. 2015) evaluates the Big Five personality traits: Extraversion, Agreeableness, Conscientiousness, Emotional Stability, and Openness to Experience. Each trait is assessed using a pair of adjectives on a five‐point Likert scale, ranging from 1 (*strongly disagree*) to 5 (*strongly agree*). The Italian version has shown satisfactory convergent validity with established Big Five measures and good test–retest reliability (Chiorri et al. 2015).

#### 2.2.4 Optimism

The Revised Life Orientation Test (LOT‐R; Scheier et al. 1994) assesses an individual's general expectation for positive future events and outcomes. It consists of six content items and four filler items, rated on a five‐point Likert scale (1 = *strongly disagree*; 5 = *strongly agree*), with higher scores reflecting greater optimism. The Italian version of the LOT‐R has shown excellent internal consistency, factorial validity, and construct validity (Chiesi et al. 2013).

#### 2.2.5 Stress

The Perceived Stress Scale (PSS‐4) includes four items that measure self‐reported mental and emotional stress experienced over the past month (Cohen et al. 1983). Participants rate their responses on a five‐point Likert scale (0 = *never*; 4 = *very often*). The Italian version of the PSS‐4 has shown strong reliability and structural validity (Mondo et al. 2022).

#### 2.2.6 Satisfaction With Life

The Satisfaction with Life Scale (SWLS; Diener et al. 1985) uses a seven‐point Likert scale (1 = *strongly disagree*; 7 = *strongly agree*) to evaluate cognitive dimensions of subjective well‐being. The Italian version of the SWLS has shown excellent internal consistency, concurrent validity, and structural validity (Diener et al. 1985; Di Fabio and Gori 2020).

### 2.3 Data Analytic Strategy

#### 2.3.1 Preliminary Item Screening

The Italian sample (*n* = 298) met established guidelines for CFA, exceeding the recommended sample‐to‐parameter ratio of 5–10 participants per estimated parameter. Prior simulation studies further suggest that models with moderate loadings (> 0.40) and adequate communalities can yield stable solutions with samples of approximately 250–300 (Wolf et al. 2013; Kline 2016). All items were examined for normality, as skewness and kurtosis values should not exceed 2 to 3 and 7 to 8, respectively (Curran et al. 1996; Finney and DiStefano 2006). Item location and item adequacy indices were calculated with Quartile of Ipsative Means (QIM), Relative Difficulty Index (RDI), and Measure of Sampling Adequacy (MSA). QIM refers to the means of items along the distribution for average values by participants and most of items should be placed in the central quartiles. RDI examines the position of the items with recommended values between 0.40 to 0.60 and 75% of values should be within that range with other items distributed evenly across the tail. MSA values below 0.50 indicate that the item may measure a different domain compared with other items. Evaluation of eigenvalues indicated a strong first eigenvalue (12.72; 43% of variance explained), followed by smaller values for the next six factors (1.88, 1.52, 1.36, 1.26, and 1.09). All subsequent eigenvalues were below 1. Analyses were conducted on FACTOR 12.03.02.

#### 2.3.2 Confirmatory Factor Analysis

Following the models proposed by Ruch and Heintz (2018), confirmatory factor analyses were performed on the global factor, six‐factor, and bifactor models using maximum likelihood estimation. Fit indices were assessed according to the criteria established by Schermelleh‐Engel et al. (2003): *χ* ^2^/df ratios of ≤ 2 and ≤ 3 were considered good and acceptable, respectively; comparative fit index (CFI) values of ≥ 0.97 and ≥ 0.95 were deemed good and acceptable, respectively; root mean square error of approximation (RMSEA) values of ≤ 0.05 and ≤ 0.08 were viewed as good and acceptable, respectively; and standardized root mean square residual (SRMR) values of ≤ 0.05 and ≤ 0.10 were categorized as good and acceptable, respectively (Hu and Bentler 1999; Schermelleh‐Engel et al. 2003). Data analyses were conducted using R: lavaan (Rosseel 2012).

#### 2.3.3 Item Response Theory

IRT provides detailed insights into item characteristics like difficulty and discrimination and ensures parameter invariance across populations. This makes IRT ideal for developing reliable and efficient assessments for short forms. Before calibrating using item response theory (IRT), the assumptions of dimensionality, local independence, and monotonicity were assessed (de Ayala 2009). Item fit under the Graded Response Model (GRM) was evaluated using S‐ *χ* ^2^ statistics (Bock and Aitkin 1981), where a nonsignificant S‐*χ* ^2^ value indicates proper item fit. The assumption of monotonicity, where the probability of endorsing an item increases with higher levels of the latent trait, was tested using R: *mokken* (Van der Ark 2007). The rest‐score function and statistical tests were used to check for monotonicity violations. After confirming unidimensionality of the scale, absence of local dependence, and monotonicity, Samejima's graded response model (GRM; Samejima 1969) was applied with marginal maximum likelihood estimation. The GRM provided item discrimination values (a) and six category threshold (bi) function values for each item. Higher discrimination values indicate better measurement of the latent trait with less measurement noise (Samejima 1969). According to Baker and Kim (2004), item discrimination cut‐off values are: ≤ 0.24 as very low, 0.25–0.64 as low, 0.65–1.34 as moderate, 1.35–1.69 as high, and ≥ 1.7 as very high. The threshold parameters (*b*_*i*_) were scaled as z‐scores (*M* = 0, SD = 1), representing the level of the latent trait needed for a 50% chance of endorsing the next response category.

#### 2.3.4 Construct Validity

Bayesian Pearson's *r* correlations were used to assess the relationships between the SHS‐PSF and validity measures. Jeffreys's (1961) Bayes Factor was employed to analyze the data by comparing a priori and posterior distributions, which allowed for the quantification of evidence supporting the alternative hypothesis versus the null hypothesis (Ly et al. 2016). According to Jeffreys's (1961) Bayes Factors, evidence supporting the alternative hypothesis is interpreted as weak (1–3), substantial (3–10), strong (10–30), very strong (30–100), or decisive (> 100) (Jarosz and Wiley 2014). All analyses were performed using a default uniform prior in JASP 0.14 (JASP Team 2020).

#### 2.3.5 Multigroup Confirmatory Factor Analysis

To assess cross‐language measurement evaluation, the equality constraints were evaluated by examining changes in the Comparative Fit Index (ΔCFI) and Root Mean Square Error of Approximation (ΔRMSEA). A ΔCFI value of ≤ 0.01 (Byrne 2001; Cheung and Rensvold 2002) and a ΔRMSEA of ≤ 0.015 indicate invariance (Chen 2007). Initially, a single‐group Confirmatory Factor Analysis (CFA) was performed to explore the factorial structure for both Italian‐ and English‐speaking samples. Metric invariance was tested by comparing the configural model with a model that constrained all item loadings to be equal across groups. This constrained model was then compared with the configural model, where item loadings were estimated freely. Scalar invariance was evaluated by imposing equal constraints on both intercepts and loadings across groups. Strict measurement invariance was assessed by also requiring that latent means be equal across groups.

#### 2.3.6 Differential Item Functioning

An ordinal logistic regression DIF approach was used to examine whether Italian‐ and English speakers had similar responses to the SHS‐PSF items (Choi et al. 2011). Uniform DIF indicates differences in the likelihood of responding to items across all levels of the trait, while nonuniform DIF shows interactions between trait levels, group, and response probabilities (Millsap and Everson 1993). Changes in pseudo *R* ^2^ values are categorized as negligible (pseudo *R* ^2^ < 0.035), moderate (0.035–0.070), or large (≥ 0.070; Gelin and Zumbo 2003). Given the tendency of *χ* ^2^ methods to be overly sensitive in detecting DIF, McFadden's pseudo *R* ^2^ > 0.035 was used as the threshold for DIF analysis (Jodoin and Gierl 2001). A 10% change in proportional *β* is considered a significant indication of uniform DIF (Crane et al. 2007).

## 3 Results

### 3.1 Descriptive Statistics

All item skewness and kurtosis values were within normal limits (|0.03|–|1.32| and |0.02|–|2.15|, respectively). All six subscales demonstrated sufficient variability (SD > 1) and the mean absolute value of skewness and kurtosis of the six humor factors was 0.27 and 0.29, respectively. Evaluation of QIM values shows 9 items in the first quartile, 12 items in the second quartile, 5 items in the third quartile, and 3 items in the fourth quartile. RDI values range from 0.19 to 0.77 (*M* = 0.59). MSA revealed that items 1 and 11 may not measure the same domain as the remaining items in the pool (Lorenzo‐Seva and Ferrando 2021). Item adequacy values are reported in Supporting Information S2.

### 3.2 Confirmatory Factor Analysis

According to the guidelines from Schermelleh‐Engel et al. (2003), the six‐factor model showed fit ranging from insufficient to acceptable, with *χ* ^2^(362) = 1109.60, *p* < 0.001, CFI = 0.85 (insufficient), TLI = 0.83 (insufficient), RMSEA = 0.08 (acceptable), and SRMR = 0.057 (acceptable). This fit was better than the one‐factor model, which had *χ* ^2^ (377) = 1749.28, *p* < 0.001, CFI = 0.72, TLI = 0.70, RMSEA = 0.08, and SRMR = 0.055. In the six‐factor model, all loadings were greater than 0.30 (all *p* < 0.001), with specific ranges for each factor: enjoyment of humor (0.36 to 0.74), laughter (0.74 to 0.77), verbal humor (0.50 to 0.84), humor under stress (0.72 to 0.86), humor in everyday life (0.55 to 0.80), and laughing at oneself (0.57 to 0.83). Correlations between the six factors ranged from 0.29 to 0.73, all statistically significant. The bifactor model also showed fit from insufficient to acceptable: *χ* ^2^(348) = 1012.06, *p* < 0.001, CFI = 0.87 (insufficient), TLI = 0.85 (insufficient), RMSEA = 0.08 (acceptable), and SRMR = 0.055 (acceptable). Overall, the fit indices indicate that while the six‐factor and bifactor models had acceptable fit based on most criteria, the CFI and TLI values were consistently insufficient across all models.

### 3.3 Item Response Theory Analysis

Before calibrating with item response theory (IRT), a dominant first factor was identified, accounting for over 20% of the variance (Reckase 1979). Additionally, the *χ* ^2^LD statistic was used to detect local dependence (Chen and Thissen 1997). IRT models require that the association between the latent trait and the probability of item endorsement increases monotonically (Reise and Rodriguez 2016). Analysis of the rest‐score function and monotonicity tests indicated that the response data exhibited a monotonic increase, with higher trait levels correlating with higher item endorsement rates. A test information function score of 10 or higher generally indicates reliability of 0.90, based on classical test theory (Cappelleri et al. 2014). The test information curve, a graphical representation of this function, is provided in Supporting Information.

The difficulty levels of items, along with the item information function curve, were examined, demonstrating that items covered a broad range across the latent trait continuum. Item discrimination values ranged from 0.32 to 2.58 (*M* = 1.61), with 2 of 29 items (items 1 and 11) showing low discrimination parameters (*a* < 0.64). Details on chi‐square fit statistics, item discrimination, and category threshold estimates are available in Supporting Information S3.

### 3.4 Reliability

Bayesian single‐test McDonald's ω reliability with 95% credible intervals (CI) is reported: enjoyment of humor (ω = 0.57, 95% CI [0.49, 0.65]), laughter (ω = 0.79, 95% CI [0.75, 0.83]), verbal humor (ω = 0.84, 95% CI [0.81, 0.86]), humor under stress (ω = 0.90, 95% CI [0.88, 0.91]), humor in everyday life (ω = 0.86, 95% CI [0.83, 0.88]), and laughing at yourself (ω = 0.85, 95% CI [0.82, 0.87]). Conditional reliability based on Bayes expected a posteriori (EAP)/overall reliability of fully informative prior oblique N‐EAP scores (ORION) reliabilities distribution was also tested (Ferrando and Lorenzo‐Seva 2016). This function provides information from the calibration stage with the prior distribution of traits and minimum square error, leading to better estimates of “true” trait prediction (Ferrando and Lorenzo‐Seva 2016). Results revealed high reliability > 0.90 across the sense of humor latent continuum from −2.66 to 2.77, indicating high reliability from low to high levels of the trait distribution.

### 3.5 Construct Validity

Bivariate Bayesian Pearson's *r* correlations of the Italian SHS‐PSF and related theoretical constructs were computed (Table 1). Sense of humor subscales showed positive associations with cheerfulness (*r* = 0.25 to 0.60, BF_10_ > 100) and five of six subscales (i.e., with the exception of enjoyment of humor) showed negative associations with bad mood (*r* = −0.19 to −0.26, BF_10_ > 100).

**TABLE 1:** External validity of sense of humor subscales with relevant theoretical constructs.

	Mean (SD)	Cheerfulness	Seriousness	Bad mood	Extraversion	Agreeableness	Conscientiousness	Emotional stability	Openness to experience	Optimism	Stress	Satisfaction with life
Reliability	—	0.89 [0.87, 0.91]	0.78 [0.74, 0.82]	0.85 [0.82, 0.88]	0.58 [0.49, 0.65]	0.26 [0.15, 0.36]	0.40 [0.30, 0.49]	0.51 [0.42, 0.59]	0.37 [0.27, 0.46]	0.64 [0.58, 0.71]	0.78 [0.74, 0.82]	0.87 [0.85, 0.89]
Enjoyment	4.60 (1.13)	0.25***	0.04	−0.14	0.10	0.11	−0.07	0.12	−0.13	0.00	−0.03	0.07
Laughter	3.93 (1.40)	0.60***	−0.10	−0.25***	0.32***	0.05	−0.10	0.19*	0.12	0.19*	−0.18	0.31***
Verbal	4.08 (1.18)	0.40***	−0.07	−0.19*	0.29***	0.08	−0.14	0.16	0.10	0.07	−0.05	0.19*
Stress	4.77 (1.31)	0.43***	−0.06	−0.26***	0.22***	0.08	−0.07	0.22***	0.14	0.16	−0.16	0.25***
Everyday	4.91 (1.06)	0.51***	−0.02	−0.26***	0.28***	0.11	0.00	0.22***	0.16	0.19*	0.14	0.30***
Laugh Self	4.86 (1.25)	0.40***	−< 0.01	−0.19*	0.27***	0.08	−0.10	0.13	0.10	0.12	−0.13	0.19*

*Note:* Reliability estimates include Bayesian Pearson's *r* for broad personality traits and Bayesian McDonald's Omega with 95% Credible Intervals for all other traits.

* BF_10_ > 10.

*** BF_10_ > 100.

With the exception of enjoyment of humor, all humor subscales revealed positive links with extraversion (*r* = 0.22 to 0.32, BF_10_ > 100). Emotional stability was positively correlated with stress and humor in everyday life (both *r* = 0.22, BF_10_ > 100). Optimism demonstrated positive associations with laughter and humor in everyday life (both *r* = 0.19, BF_10_ > 10). All five subscales, except enjoyment of humor, were positively associated with satisfaction with life (*r* = 0.19 to 0.32, BF_10_ > 10). No associations were found between sense of humor subscales and seriousness, agreeableness, openness to experience conscientiousness, or stress.

### 3.6 Measurement Invariance and Differential Item Functioning

The Canadian sample was larger than the Italian sample due to differences in recruitment feasibility across countries. To reduce potential bias from this imbalance, analyses were first conducted within each cultural group, and measurement invariance testing was applied before making cross‐cultural comparisons.

Measurement invariance with multigroup CFA of the SHS‐PSF across Italian‐ and English‐speaking samples was examined. Fit indices and the change between each subsequent restriction (loadings, intercepts, residuals, and means) of different models are reported in Table 2. The CFI and RMSEA change were > |0.01| and > |0.015|, respectively, for the scalar invariance models, suggesting differential intercepts across Italian and English versions.

**TABLE 2:** Multigroup confirmatory factor analysis for Italian and English versions of the sense of humor scale parallel version short form.

	*χ* ^2^	∆*χ* ^2^	CFI	∆CFI	RMSEA	∆RMSEA
Configural	2709.89 (724)		0.882		0.067	
Metric	2854.16 (747)	144.27***	0.875	0.007	0.068	0.001
Scalar	4074.24 (770)	1220.08***	0.804	0.054	0.084	0.016
Strict	4655.32 (799)	581.08***	0.771	0.033	0.089	0.005

Abbreviations: CFI, Comparative Fit Index; RMSEA, Root Mean Square Error of Approximation.

*** *p* < 0.001.

Based on logistic regression DIF approaches, four items had uniform DIF using the beta criterion (*β* > 0.10) and one item showed uniform DIF using McFadden's pseudo *R* ^2^ > 0.035. These items showed uniform DIF using the beta criterion: 3 (“I often tell jokes”), 4 (“I have no trouble poking fun at my physical imperfections”), 5 (“My sense of humor rarely abandons me under stress.”), and 14 (“I enjoy funny sketches”). McFadden's pseudo *R* ^2^ was largest at 0.17 in pseudo *R* ^2^ between Models 1 and 2 for item 3, suggesting a large uniform differential item functioning effect. All remaining items showed McFadden pseudo *R* ^2^ values < 0.035, suggesting that the overall impact of these differences was minimal. Items flagged for DIF were plotted as item and test characteristic curves.

## 4 Discussion

The SHS‐PSF is a tool developed to evaluate humor skills among Italian populations, aiming to broaden its application in measuring humor‐related behaviors in Italy. Analysis of item‐level characteristics, factorial validity, and measurement invariance has shown that the Italian version of the SHS‐PSF generally exhibits psychometric properties similar to those reported in earlier studies, although there are some concerns about fit indices for CFI and TLI (Heintz et al. 2021; Ruch and Heintz 2018). The primary objective was to assess the reliability and validity of this newly adapted Italian measure. Examination of the item pool's dimensionality revealed that both a general sense of humor factor and six distinct humor skills factors could be identified. This indicates that the SHS‐PSF includes a general humor component present across all items, reflecting one of six humor skills. Although the six‐factor and bifactor models demonstrated acceptable fit, the CFI and TLI values were less satisfactory. The CFI and TLI are incremental fit indices that show how much the hypothesized model improves over the baseline model, while RMSEA is an absolute fit index that indicates how close the model is to an ideal model (Xia and Yang 2019). These findings align with previous results for the SHS, which also showed acceptable absolute fit but weaker incremental fit indices (Heintz et al. 2021; Ruch and Heintz 2018). It may be challenging to determine which model is empirically superior, and future research could focus on revising items to improve incremental fit indices.

The comparable fit of the six‐factor and bifactor models suggests that both structures capture important aspects of the data, even though neither achieves optimal fit. The bifactor model supports the presence of a unidimensional overarching factor, sense of humor, while the six‐factor model highlights the distinct but related dimensions of enjoyment of humor, laughter, verbal humor, finding humor in everyday life, laughing at oneself, and humor under stress. The moderate to strong correlations among these factors indicate that they share common variance, consistent with the existence of a global humor construct, while still retaining unique variance that reflects the multifaceted nature of humor. This pattern underscores the theoretical plausibility of both perspectives and suggests that future scale development may benefit from refining items to improve model fit while maintaining sensitivity to both global and domain‐specific aspects of humor.

Samejima's graded response model analysis and MSA values suggested that items 1 (“I generally look for sitcoms or other funny programs to watch on TV”) and 11 (“When I go to the movies, my preference is generally to see a good comedy”) might exhibit notable measurement noise compared to other items. Reliability indices were satisfactory for five out of six humor skills, except for enjoyment of humor, which aligns with previous findings (Ruch and Heintz 2018). The preference for comedy as a genre may not fully capture the frequency or intensity of an individual's enjoyment of humor in everyday situations. Future research might explore whether including items that address individuals' thoughts, feelings, and behaviors in response to enjoyable content could improve reliability.

The second aim was to establish construct validity by examining relationships with conceptually relevant constructs. As expected a priori, sense of humor subscales correlated positively with cheerfulness and negatively with bad mood, consistent with the temperamental basis of humor. Likewise, positive associations with extraversion and optimism, and with life satisfaction, align with theoretical predictions that humor skills foster positive emotions, sociability, and well‐being (McGhee 1996; Ruch and Hofmann 2012; Ruch and Heintz 2018). The absence of associations with traits such as agreeableness, openness, conscientiousness, and seriousness further supports discriminant validity, indicating that the SHS‐PSF captures humor‐related traits specifically rather than broad personality dimensions.

While Lydon and McDermut (2021) found that the SHS was linked to extraversion, this study found that all six humor habits were associated with both cheerfulness and extraversion. With higher correlations between SHS traits and cheerfulness, these results support the idea that cheerfulness is a more specific personality trait related to humor behaviors than extraversion (Ruch and Hofmann 2012). Interestingly, enjoyment of humor was not linked to extraversion, which aligns with Lydon and McDermut's (2022) findings. They suggested that some items in the enjoyment of humor subscale might be outdated. For instance, preferences for TV shows or comics might not reflect current entertainment trends like internet streaming platforms (e.g., YouTube) or forums (e.g., Reddit). Additionally, enjoyment of humor might capture humor appreciation or comprehension rather than humor expression as a personality trait. While the present study included broad personality traits and temperamental humor, future studies would benefit from correlations of this measure with other humor measures validated in Italy. Future research could investigate whether updating the assessment of enjoyment of humor to include more contemporary forms of entertainment could impact its structural and criterion validity.

Although McGhee (1996) proposed that seriousness might prevent people from engaging in humorous behaviors, there was no evidence linking seriousness with any of the six humor habits. Recent research has indicated that while cheerfulness is associated with using humor under stress, low seriousness might only lead to more frequent use of humor under stress when cheerfulness is also low (Lau et al. 2022b). Additionally, Lau et al. (2019) found that low cheerfulness was linked to increased resilience in individuals with higher levels of seriousness. These findings suggest that seriousness may influence humor habits primarily in the context of low cheerfulness. Future research should explore whether the interaction between these traits could predict different psychosocial outcomes related to humor use.

The third objective was to examine cultural invariance between the Italian and English versions of the SHS‐PSF. DIF analyses revealed that 4 out of 29 items exhibited uniform DIF, while the rest performed similarly across Italian‐ and English speakers. Notably, item 3 (“I often tell jokes”) showed significant DIF, with Italian speakers finding it more challenging. Multigroup CFA results supported metric invariance but not scalar invariance. These results suggest that the differences between the Italian and English versions of the SHS‐PSF might be due to methodological issues (such as item interpretation or translation) rather than true differences. Additionally, English‐speaking participants were generally younger, whereas Italian‐speaking participants had more diverse ages and educational backgrounds. Future research should aim to recruit comparable samples (in terms of age, socioeconomic status, and education) to better assess the cross‐cultural validity of the SHS‐PSF.

The DIF findings may, in part, reflect cultural differences in how humor is expressed and valued rather than flaws in measurement. Prior research also shows Italians rate sexual humor more positively but find jokes less funny overall than Germans (Ruch and Forabosco 1996), highlighting differences in humor appreciation that may underlie item‐level variation. More recent work has shown that humor in Italy is also shaped by context and stress, such as during the COVID‐19 pandemic (Bischetti et al. 2021; Canestrari et al. 2021). These cultural patterns suggest that the observed DIF effects may capture meaningful differences in humor expression across societies rather than measurement deficiencies.

Several limitations of this study highlight areas for further research. First, participants were recruited online and the sense of humor scales and construct validity indicators were collected simultaneously. Socially desirable traits (e.g., extraversion, cheerfulness, optimism, emotional stability, life satisfaction) often correlate with each other and negatively with undesirable traits (e.g., bad mood, stress). Future research should include behavioral measures to address the limitations of relying solely on questionnaire‐based data. A longitudinal design could provide insights into causal relationships by collecting criterion validity variables at different time points. Additionally, if humor can be trained, data collected over multiple sessions might better track changes over time. Second, it is challenging to determine whether differences observed in cultural invariance testing are due to language versions or age groups. Future studies should match Italian and English‐speaking participants by age, gender, and socioeconomic status. Third, while efforts were made to ensure the quality of the Italian online data collection, future research should include quality checks on the data. Fourth, the sample had fewer men than women, which prevented gender invariance analyses. Future studies should aim for a more balanced gender distribution. Fifth, a limitation of the present study is the use of the Ten Item Personality Measure (TIPI; Gosling et al. 2003; Chiorri et al. 2015), which, while efficient, provides only a brief assessment of the Big Five traits. As such, the depth of construct validation is constrained, and stronger conclusions may be possible in future studies that employ more comprehensive personality measures. Finally, construct validity was assessed using only one humor temperament measure (the state–trait cheerfulness inventory). Future research should explore criterion validity with additional humor‐related variables, such as benevolent and corrective humor.

Future research should build on these findings by addressing several key areas. First, the psychometric properties of the four items that demonstrated uniform DIF between the Italian and English versions warrant re‐examination to determine whether these items require refinement or replacement. Second, it will be important to investigate whether the same DIF patterns appear when the Italian version is compared with English‐speaking populations outside of Canada, in order to disentangle potential linguistic artifacts from cultural differences. Finally, although the bifactor model showed better fit than the unidimensional model, its indices still fell below conventional adequacy thresholds; future work should further explore bifactor structures to evaluate whether a general humor factor can be meaningfully interpreted alongside the six humor dimensions. Together, these avenues of inquiry will help refine the scale and clarify the extent to which humor measurement is consistent across languages and cultures.

Overall, the Italian SHS‐PSF is a reliable and valid psychometric tool, albeit with some mixed results that require further studies. Utilizing sense of humor measures in Italy could improve our understanding of humor across various populations and contexts.

## Author Contributions

**Chloe Lau:** contributed to conceptualization, data collection, data analysis, formal writing, preparation of the original draft, and review and editing. **Willibald Ruch:** contributed to conceptualization, original draft preparation, and review and editing. **Sonja Heintz:** contributed to original draft preparation and review and editing. **Lena C. Quilty:** provided supervision, contributed to original draft preparation, and review and editing. **Francesco Bruno:** contributed to the translation of the measure, original draft preparation, and review and editing. **Donald Saklofske:** contributed to data collection and review and editing. **Francesca Chiesi:** contributed to data collection, translation of the measure, supervision, and review and editing.

## Ethics Statement

All procedures performed in studies involving human participants were in accordance with the ethical standards of the University of Western Ontario and with the 1964 Helsinki Declaration and its later amendments or comparable ethical standards.

## Consent

This study was conducted according to the American Psychological Association (APA) and National Association of Psychology ethical standards for the treatment of human subjects. Data collection was anonymous and involved no identifying information and no medical treatment. Participants were informed that their participation was voluntary, that they could leave the study at any time, and that their data would be treated anonymously. In addition, they were informed that by starting the survey they would be considered to have read and accepted the informed consent.

## Conflicts of Interest

The authors declare no conflicts of interest.

## Supporting information


**Data S1:** sjop70049‐sup‐0001‐Supinfo1.docx.

**Data S2:** sjop70049‐sup‐0002‐Supinfo2.docx.

**Data S3:** sjop70049‐sup‐0003‐Supinfo3.docx.

**Data S4:** sjop70049‐sup‐0004‐Supinfo4.docx.

## Data Availability

The data presented in this study are available on request from the corresponding author. The data are not publicly available.
