# Supplementary material for: Psychometric Analysis and Cross‐Cultural Comparisons of the Italian and English Sense of Humor Scale Parallel Version Short Form
Source: Scand J Psychol. 2025 Dec 15;67(3):686–96. doi: 10.1111/sjop.70049 (PMC13159503; doi:10.1111/sjop.70049)
Supplement: Supplementary file 1 — Data S1: sjop70049‐sup‐0001‐Supinfo1.docx. [file SJOP-67-686-s002.docx]

**Sense of Humor Parallel Version Short Form**


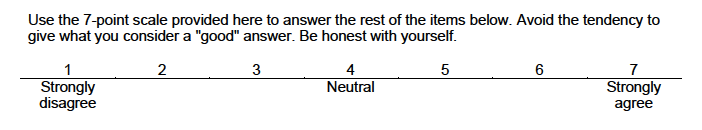


| Item Code Original (Short Form) | English | Italian |
| --- | --- | --- |
| SHS_1 (1) | I generally look for sitcoms or other funny programs to watch on TV. | Di solito cerco serie tv o altri programmi divertenti da guardare in televisione. |
| SHS_2 (2) | I have a good belly laugh many times each day. | Rido a crepapelle molte volte al giorno |
| SHS_3 (3) | I often tell jokes. | Racconto spesso barzellette |
| SHS_5 (4) | I have no trouble poking fun at my physical imperfections | Non ho alcun problema nel fare autoironia sulle mie imperfezioni fisiche |
| SHS_6 (5) | My sense of humor rarely abandons me under stress. | Il mio senso dell’umorismo raramente mi abbandona anche se sono sotto stress |
| SHS_8 (6) | I have a heartier, more robust laugh than most people. | Rido di gusto e più forte della maggior parte delle persone |
| SHS_9 (7) | I often tell funny stories. | Racconto spesso storie divertenti |
| SHS_10 (8) | I often find humor in things that happen at home | Trovo spesso divertenti alcune cose che accadono a casa |
| SHS_11 (9) | I often find humor in my own embarrassing incidents or personal blunders. | Spesso trovo divertenti le mie figure imbarazzanti o gli errori grossolani che posso commettere |
| SHS_12 (10) | I often use my sense of humor to control the effect of stress on my mood | Spesso uso il mio humor per contenere l’effetto dello stress sul mio stato d’animo |
| SHS_13 (11) | When I go to the movies, my preference is generally to see a good comedy. | Quando si tratta di scegliere un film, generalmente opto per una commedia. |
| SHS_15 (12) | I often create my own spontaneous puns. | Creo spesso spontaneamente dei giochi di parole divertenti |
| SHS_22 (13) | I often share with others the funny incidents I observe, or that happen to me. | Spesso condivido con gli altri situazioni divertenti che osservo o che succedono a me. |
| SHS_25 (14) | I enjoy funny sketches. | Mi piacciono gli sketch divertenti |
| SHS_27 (15) | I like to engage in conversations with witty comments. | Mi piace impegnarmi in conversazioni con commenti spiritosi |
| SHS_28 (16) | I can see funny things in many activities. | Riesco a vedere cose divertenti in molte attività. |
| SHS_29 (17) | I can laugh about myself. | Posso ridere di me stesso |
| SHS_30 (18) | My sense of humor is a good way to relieve stress. | Il mio senso dell'umorismo è un buono strumento per alleviare lo stress. |
| SHS_31 (19) | On the Internet, I am looking for  content that amuses me. | Su internet cerco contenuti che mi facciano divertire |
| SHS_33 (20) | I often describe events as witty anecdotes. | Descrivo spesso i fatti come aneddoti spiritosi. |
| SHS_34 (21) | I often discover funny occurrences in the everyday routine | Spesso scopro cose divertenti nella routine quotidiana |
| SHS_35 (22) | If something embarrassing  happens to me, I can laugh about  it. | Se mi succede qualcosa di imbarazzante, riesco a riderci sopra |
| SHS_36 (23) | My humor helps me to stay serene when my life is hectic. | Il mio umorismo mi aiuta a rimanere sereno quando la mia vita è frenetica. |
| SHS_38 (24) | I often laugh till I get tears in the eyes. | Rido spesso fino a farmi venire le lacrime occhi. |
| SHS_39 (25) | I often make funny comments. | Faccio spesso commenti buffi |
| SHS_40 (26) | I often notice amusing aspects in other people's behaviors. | Spesso noto aspetti divertenti nei comportamenti delle persone. |
| SHS_46 (27) | I more often notice funny things in everyday life than other people do. | Noto cose divertenti nella vita di tutti i giorni. più spesso di quanto facciano gli altri |
| SHS_47 (28) | If someone makes a joke about me, I can laugh about it. | se qualcuno fa una battuta su di me, riesco a riderne. |
| SHS_48 (29) | Even in difficult situations I don't lose my sense of humor. | Anche in situazioni difficili non perde il mio senso dell’umorismo. |

**Calculation for items:**

| SHS_Enjoy | (SHS_1_N01 + SHS_11_N13 + SHS_14_N25 + SHS_19_N31)/4 |
| --- | --- |
| SHS_Laughter | (SHS_2_N02 + SHS_6_N08 + SHS_24_N38)/3 |
| SHS_Verbal | (SHS_3_N03 + SHS_7_N09 + SHS_12_N15 + SHS_15_N27 + SHS_20_N33 + SHS_25_N39)/6 |
| SHS_HumorStress | (SHS_5_N06 + SHS_10_N12 + SHS_18_N30 + SHS_23_N36 + SHS_29_N48)/5 |
| SHS_HumorEveryday | (SHS_8_N10 + SHS_13_N22 + SHS_16_N28 + SHS_21_N34 + SHS_26_N40 + SHS_27_N46)/6 |
| SHS_LaughSelf | (SHS_4_N05 + SHS_9_N11 + SHS_17_N29 + SHS_22_N35 + SHS_28_N47)/5 |
