# Supplementary material for: Psychometric Analysis and Cross‐Cultural Comparisons of the Italian and English Sense of Humor Scale Parallel Version Short Form
Source: Scand J Psychol. 2025 Dec 15;67(3):686–96. doi: 10.1111/sjop.70049 (PMC13159503; doi:10.1111/sjop.70049)
Supplement: Supplementary file 2 — Data S2: sjop70049‐sup‐0002‐Supinfo2.docx. [file SJOP-67-686-s004.docx]

**Supporting Information S2**

**Item Adequacy Indices**

| Items | QIM | RDI | Normed MSA |
| --- | --- | --- | --- |
| 3 | 1 | .19 | .90 |
| 12 | 1 | .43 | .90 |
| 6 | 1 | .44 | .93 |
| 7 | 1 | .46 | .92 |
| 11** | 1 | .46 | .71 |
| 24 | 1 | .49 | .90 |
| 2 | 1 | .53 | .94 |
| 27 | 1 | .54 | .95 |
| 9 | 1 | .55 | .92 |
| 19 | 2 | .57 | .88 |
| 29 | 2 | .59 | .95 |
| 23 | 2 | .60 | .95 |
| 21 | 2 | .60 | .94 |
| 20 | 2 | .62 | .94 |
| 1** | 2 | .62 | .76 |
| 22 | 2 | .62 | .92 |
| 10 | 2 | .63 | .92 |
| 25 | 2 | .63 | .95 |
| 8 | 2 | .64 | .94 |
| 5 | 2 | .64 | .94 |
| 28 | 2 | .65 | .93 |
| 4 | 3 | .67 | .89 |
| 16 | 3 | .67 | .95 |
| 26 | 3 | .68 | .93 |
| 18 | 3 | .69 | .95 |
| 17 | 3 | .72 | .91 |
| 15 | 4 | .75 | .94 |
| 14 | 4 | .75 | .86 |
| 13 | 4 | .78 | .91 |
