# Supplementary material for: Psychometric Analysis and Cross‐Cultural Comparisons of the Italian and English Sense of Humor Scale Parallel Version Short Form
Source: Scand J Psychol. 2025 Dec 15;67(3):686–96. doi: 10.1111/sjop.70049 (PMC13159503; doi:10.1111/sjop.70049)
Supplement: Supplementary file 3 — Data S3: sjop70049‐sup‐0003‐Supinfo3.docx. [file SJOP-67-686-s001.docx]

**Supporting Information S3**

Chi Square Fit Statistic, Item Discrimination, and Category Threshold Estimates for Samejima’s Graded Response Model

| Item | S_χ^2^ (df) | a | b_1_ | b_2_ | b_3_ | b_4_ | b_5_ | b_6_ |
| --- | --- | --- | --- | --- | --- | --- | --- | --- |
| 1 | 85.16 (87) | .39 | -6.01 | -4.46 | -3.14 | -1.67 | 1.04 | 4.79 |
| 2 | 88.85 (92) | 1.45 | -2.55 | -1.57 | -.70 | .24 | 1.23 | 2.18 |
| 3 | 79.34 (74) | 1.00 | -.16 | .99 | 1.74 | 2.52 | 3.54 | 5.11 |
| 4 | 95.77 (92) | 1.03 | -3.93 | -2.47 | -1.70 | -.98 | .08 | 1.82 |
| 5 | 90.58 (81) | 1.96 | -2.61 | -1.55 | -1.08 | -.43 | .28 | 1.27 |
| 6 | 90.07 (95) | 1.39 | -1.78 | -.83 | -.19 | .72 | 1.49 | 2.42 |
| 7 | 92.25 (100) | 1.77 | -1.52 | -.82 | -.24 | .38 | 1.09 | 2.13 |
| 8 | 77.71 (84) | 1.38 | -2.95 | -2.04 | -1.37 | -.56 | .47 | 1.66 |
| 9 | 59.80 (79) | 1.53 | -2.04 | -1.25 | -.65 | -.23 | .79 | 1.87 |
| 10 | 118.11 (93) | 1.56 | -3.48 | -1.85 | -1.07 | -.43 | .52 | 1.39 |
| 11 | 105.68 (97) | .32 | -5.92 | -2.96 | -.44 | 2.07 | 4.14 | 7.21 |
| 12 | 92.91 (101) | 1.00 | -1.98 | -.71 | .11 | .79 | 1.68 | 2.86 |
| 13 | 71.83 (68) | 1.35 | -4.06 | -3.21 | -2.57 | -1.74 | -.54 | 1.12 |
| 14 | 84.42 (83) | 1.06 | -4.84 | -3.34 | -2.55 | -1.64 | -.37 | 1.09 |
| 15 | 81.55 (69) | 1.84 | -3.22 | -2.55 | -1.86 | -1.18 | -.18 | .88 |
| 16 | 57.45 (64) | 2.22 | -3.11 | -2.12 | -1.48 | -.57 | .25 | 1.39 |
| 17 | 69.35 (71) | 1.96 | -3.34 | -2.22 | -1.51 | -.86 | -.13 | 1.07 |
| 18 | 67.59 (69) | 2.57 | -2.94 | -1.93 | -1.24 | -.56 | .17 | .98 |
| 19 | 106.53 (95) | .85 | -2.96 | -2.00 | -1.33 | -.34 | .94 | 2.78 |
| 20 | 85.06 (79) | 2.11 | -2.58 | -1.63 | -.92 | -.31 | .43 | 1.42 |
| 21 | 82.50 (73) | 2.07 | -3.02 | -1.76 | -1.08 | -.21 | .76 | 1.76 |
| 22 | 87.71 (82) | 1.87 | -2.29 | -1.61 | -1.02 | -.42 | .42 | 1.54 |
| 23 | 61.77 (82) | 2.32 | -2.53 | -1.69 | -.78 | -.14 | .61 | 1.37 |
| 24 | 100.00 (98) | 1.22 | -2.37 | -1.17 | -.41 | .46 | 1.54 | 2.41 |
| 25 | 66.04 (71) | 2.58 | -2.35 | -1.62 | -1.06 | -.43 | .49 | 1.42 |
| 26 | 58.83 (64) | 1.65 | -3.85 | -2.46 | -1.68 | -.85 | .42 | 1.71 |
| 27 | 57.44 (71) | 2.37 | -2.14 | -1.37 | -.65 | .15 | .88 | 1.85 |
| 28 | 86.63 (76) | 1.77 | -2.90 | -2.01 | -1.24 | -.57 | .36 | 1.63 |
| 29 | 100.37 (78) | 2.09 | -2.74 | -1.43 | -.79 | -.11 | .68 | 1.55 |
