# Supplementary material for: Psychometric Analysis and Cross‐Cultural Comparisons of the Italian and English Sense of Humor Scale Parallel Version Short Form
Source: Scand J Psychol. 2025 Dec 15;67(3):686–96. doi: 10.1111/sjop.70049 (PMC13159503; doi:10.1111/sjop.70049)
Supplement: Supplementary file 4 — Data S4: sjop70049‐sup‐0004‐Supinfo4.docx. [file SJOP-67-686-s003.docx]

**Supporting Information S4**

*Test information function for the Italian Version of the Sense of Humor Parallel Version Short Form*


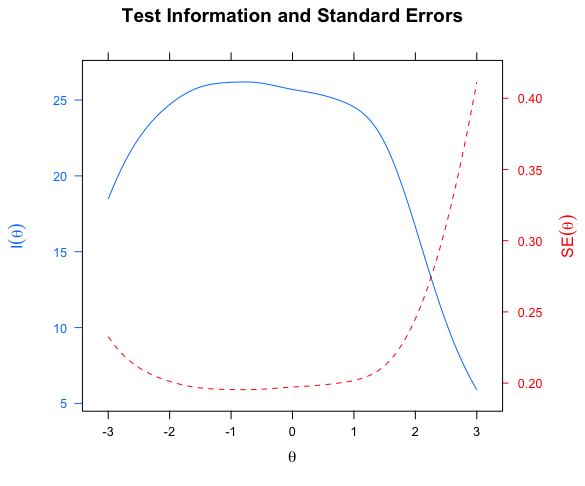


*Note*. Latent trait (y) is shown on the horizontal axis, and the amount of information (solid line) and the standard error (dotted line) yielded by the test at any trait level are shown on the vertical axis.
